# Recent Advances in Mechanobiology‐Mediated In Situ Tissue Engineering

**DOI:** 10.1155/ijbm/7100178

**Published:** 2026-07-15

**Authors:** Henry Agbe, Bright N. Jaato, Dominic A. Dadzie, Benjamin Mensah Frimpong, Prudent M. Mensah, Michael K. Appiah, David Dodoo-Arhin, Alexandre Kabla

**Affiliations:** ^1^ Department of Materials and Metallurgy Engineering, College of Engineering, Kwame Nkrumah University of Science and Technology, PMB University Post Office, KNUST, Kumasi, Ghana, knust.edu.gh; ^2^ KNUST Center of Excellence for Biomaterials and Tissue Engineering Research-KCEBTER, PMB University Post Office, KNUST, Kumasi, Ghana, knust.edu.gh; ^3^ KNUST Center for Engineering Materials Research, Kumasi, AK-448-7038, Ghana; ^4^ Center for Nanointegration Duisburg-Essen (CENIDE), Carl-Benz Street 199, Duisburg, 47057, Germany; ^5^ Department of Materials Science & Engineering, University of Ghana, P.O. Box LG 77 Legon, Accra, Ghana, ug.edu.gh; ^6^ Engineering Department, University of Cambridge, Cambridge, CB2 1PZ, UK, cam.ac.uk

**Keywords:** 3D scaffolds, biomimetics, in situ regeneration, mechanobiology, scaffolds, tissue engineering

## Abstract

Tissue engineering aims to repair, replace, or regenerate damaged tissues by integrating principles of biology, engineering, and material science. Traditional ex vivo strategies, involving prefabricated cell/scaffold constructs followed by implantation, have shown promise but face significant limitations, including poor vascularization, immune rejection, high costs, and clinical translation challenges. These limitations have driven the emergence of in situ tissue engineering, which harnesses the body’s intrinsic regenerative capacity by recruiting endogenous stem or progenitor cells to sites of injury for repair. A key requirement for successful in situ regeneration is the design of biomimetic three‐dimensional scaffolds capable of delivering bioactive molecules such as growth factors and cytokines in a controlled and spatiotemporal manner. In addition to biochemical cues, mechanobiology plays a central role by regulating cell adhesion, migration, proliferation, and differentiation through mechanotransduction pathways involving cytoskeletal remodeling, extracellular matrix (ECM) dynamics, and nuclear signaling. This review highlights mechanobiology‐mediated strategies, scaffold designs, and applications for hard and soft tissue repair, as well as challenges and future directions in regenerative medicine.

## 1. Introduction

Tissue engineering has emerged as a transformative strategy to repair, replace, and regenerate damaged tissues by integrating principles of biology, engineering, and material science [1, 2]. Traditionally, approaches have relied on ex vivo manipulation of cells and scaffolds, followed by implantation. While these methods have shown promise, they face challenges, such as limited vascularization, immune rejection, high costs, and difficulties in clinical scalability [3–5]. These limitations have spurred growing interest in in situ tissue engineering, an approach that harnesses the body’s intrinsic regenerative capacity by recruiting endogenous stem cells or tissue‐specific progenitor cells from their niche to target or injury sites of interest for regeneration [6].

A critical determinant of successful in situ regeneration is the ability to encapsulate bioactive molecules (growth factors, cytokines, and small molecules) in a biomimetic three‐dimensional (3D) scaffold for subsequent release, unlocking the body’s regeneration capability within the dynamic native microenvironment. Beyond biochemical signaling, mechanobiology (the study of how cells sense, interpret, and respond to mechanical cues) has emerged as a pivotal driver of tissue development and repair [7, 8]. Mechanical cues regulate a wide array of cellular functions, including adhesion, migration, proliferation, and lineage specification, through mechanotransduction pathways that integrate: (a) cytoskeletal dynamics; (b) extracellular matrix (ECM) remodeling; and (c) nuclear signaling [9–11]. Harnessing these mechanobiological tools offers a powerful means to engineer biomaterial 3D scaffolds that not only provide structural support but also deliver instructive mechanical and biochemical cues to guide in situ tissue formation [12, 13].

Recent advances in biomaterials, fabrication technologies, and bioinspired design have enabled the creation of scaffolding systems that actively mimic the natural ECM to engage mechanobiological pathways for in situ tissue regeneration. These innovations are redefining in situ tissue engineering by promoting vascularization, enhancing integration with host tissue, and enabling spatiotemporal control of regenerative processes for effective clinical outcomes and translations [14, 15].

Note that mechanobiological design spans three integrated mechanical length scales: (A) cell–matrix interface metrics—where, for instance, ECM stiffness (kPa) and adhesion‐level geometry (e.g., ligand spacing and focal adhesion [FA] maturation) stimulate integrin mechanosensing, cytoskeletal tension, and early lineage specification; (B) where porosity‐dependent scaffold apparent properties (such as compressive modulus, toughness, permeability, and interconnectivity) lead to cell infiltration, migration, and scaffold‐level load distribution; and (C) where organ‐level functional mechanics (such as Young’s modulus [MPa–GPa], fatigue resistance, and viscoelasticity under physiological loading) lead to long‐range tissue stability, functional integration, and performance in load‐bearing environments. Overall, such mechanobiological design integrates mechanical signal transduction from the nanoscale level, through to the cellular level, to the tissue level, and eventually to the organ level, thus supporting a coherent, design‐oriented interpretation of mechanobiology in in situ tissue engineering.

This review highlights recent advances in mechanobiology‐mediated in situ tissue engineering, with particular emphasis on mechanobiology fundamentals, mechanotransduction pathways, ECM mimetic bioactive scaffold designs, and emerging applications across hard and soft tissue regeneration. We also discuss current challenges, limitations, and future directions, underscoring the potential of this rapidly evolving field to transform regenerative medicine.

## 2. Fundamentals of Mechanobiology

Mechanobiology is the interdisciplinary study of how mechanical forces (such as tension, compression, shear stress, and matrix stiffness) and topographical features influence cellular behavior and tissue function. Drawing from biology, physics, materials science, and engineering, it explores how cells sense their physical environment and convert mechanical cues into biochemical signals that regulate gene expression, protein synthesis, proliferation, differentiation, migration, and apoptosis [1, 2].

Unlike chemical signals, mechanical cues are highly context‐dependent, and they vary across tissues, evolve over time, and operate across multiple spatial and temporal scales. From nanometer‐level to matrix stiffness, to organ‐level forces, and from rapid ion channel activation to long‐term changes in tissue architecture, these cues shape cellular behavior in dynamic and tissue‐specific ways. By decoding this mechanical language, mechanobiology provides a framework for designing responsive biomaterials and scaffolds, especially within in situ tissue engineering, where mechanical cues can be harnessed to guide endogenous tissue regeneration in a targeted and transformative manner [7, 8].

### 2.1. Types of Mechanical Cues

Cells are constantly exposed to a range of mechanical cues in their native microenvironments and within engineered systems. These cues, acting across various spatial and temporal scales, influence cell adhesion, migration, proliferation, differentiation, and remodeling. Understanding these mechanical stimuli is critical for designing biomaterials and scaffolds that support both tissue structure and guide regenerative outcomes in in situ tissue engineering [16, 17]. Below, we summarize the principal types of mechanical cues and their biological significance.

#### 2.1.1. Shear Stress

Shear stress refers to the tangential force generated by fluid flow across the surface of cells or tissues. In vivo plays a critical role in vascular and lymphatic systems, where endothelial cells are constantly exposed to shear forces. These cues regulate endothelial alignment, nitric oxide production, and anti‐inflammatory signaling, contributing to vascular homeostasis [18, 19]. Specifically, laminar shear stress activates mechanosensitive transcription factors, such as Kruppel‐like factor 2 (KLF2) and nitric oxide synthase (eNOS), promoting anti‐inflammatory and antithrombotic phenotypes [18, 20]. Conversely, disturbed or oscillatory shear is associated with endothelial dysfunction and the initiation of atherogenic processes [21, 22]. In engineered environments, shear stress is often replicated using microfluidic platforms or flow bioreactors to enhance the functional maturation of vascular and epithelial tissues [23], making it an essential consideration in the design of biomimetic systems for in situ regeneration.

#### 2.1.2. Tensile Strain

Tensile strain involves stretching forces that elongate cells and tissues. It is physiologically prevalent in load‐bearing structures, such as muscles, tendons, ligaments, and skin, where tissues undergo cyclic strain during daily movements and mechanical loading [24]. Tensile strain induces cell alignment in the direction of stretch and reorganizes the actin cytoskeleton to withstand applied forces [25]. It also stimulates the production of ECM proteins, particularly collagen types I and III, and elastin, in fibroblasts and smooth muscle cells, contributing to tissue remodeling and mechanical resilience [26, 27]. In tissue engineering, the application of cyclic tensile strain in bioreactor systems enhances the structural and functional properties of engineered tissue constructs (such as skeletal muscle, tendons, and ligaments), improving their alignment, strength, and integration potential [28, 29]. From an in situ tissue engineering perspective, integrating tensile strain as a mechanobiological cue offers a biomimetic strategy to guide cellular behavior and tissue regeneration directly within the host microenvironment. By replicating physiological mechanical loading, tensile strain can activate endogenous repair mechanisms, stimulate cell alignment and matrix deposition, and facilitate the functional integration of engineered scaffolds with native tissues. Thus, in situ tissue engineering supports the development of regenerative therapies that leverage the body’s own mechanical environment to orchestrate more effective and durable healing processes.

#### 2.1.3. Compressive Stress

Compressive stress refers to perpendicular forces that push and compact tissues, and it is a primary mechanical input in load‐bearing organs, such as articular cartilage, bone, and intervertebral disks. These tissues are subjected to frequent compressive loads during daily activities, such as walking, lifting, and maintaining posture [30]. Moderate levels of compressive stress are essential for chondrocyte function, as they stimulate ECM production and promote chondrogenic differentiation, thereby maintaining cartilage homeostasis [31]. Additionally, the gut experiences moderate tensile and compressive stresses as peristaltic waves propel the stool through the colon. In contrast, excessive or unregulated compression can lead to cell apoptosis, matrix degradation, and inflammatory responses, all of which contribute to the progression of degenerative joint diseases, such as osteoarthritis (OA) [32, 33]. To replicate these mechanical environments in vitro, compression chambers and dynamic bioreactors have been developed to apply controlled mechanical loads to tissue‐engineered constructs. These systems not only support the formation of cartilage‐ and bone‐like tissues from stem cells but also enhance the mechanical integrity and functional organization of scaffolds intended for in situ regeneration [34].

#### 2.1.4. Hydrostatic Pressure

Hydrostatic pressure is a uniform compressive force exerted by fluids surrounding tissues, particularly in enclosed or pressurized spaces, such as joints and interstitial compartments. It plays an important physiological role in maintaining tissue hydration, nutrient transport, and osmotic balance, especially in cartilage and synovial joints [35, 36]. In mechanobiology, hydrostatic pressure regulates ion transport, modulates chondrocyte function, and promotes ECM deposition. Sustained application of physiological hydrostatic pressure has been shown to enhance cell viability and cartilage‐specific matrix synthesis. Hydrostatic bioreactors are thus frequently employed in cartilage tissue engineering to simulate the native pressurized environment and improve in situ regeneration outcomes [37].

#### 2.1.5. Matrix Stiffness

Matrix stiffness is defined by the elastic modulus of the ECM or a substrate. It is a key mechanical property that influences how cells adhere, spread, and differentiate. Different tissues in the body exhibit a wide stiffness range, from soft brain tissue (∼0.1–1 kPa) to stiff bone (> 10 GPa). Cells detect stiffness through integrin‐mediated FAs and respond via activation of downstream signaling pathways, including the mechanosensitive Yes‐associated protein/transcriptional coactivator with PDZ‐binding motif (YAP/TAZ) axis [9]. Importantly, substrate stiffness plays a pivotal role in directing the fate of stem cells. For instance, soft matrices promote neurogenesis, intermediate stiffness supports myogenesis, whereas rigid matrices induce osteogenesis [9]. In scaffold design, stiffness can be tuned to match the target tissue, providing appropriate mechanical signals for lineage‐specific differentiation and functional integration during in situ tissue repair [38].

#### 2.1.6. Topography and Geometry

Topography and geometry are fundamental structural features that influence cellular behavior and tissue regeneration. While topography refers to the micro‐ and nanoscale surface features of a material (such as fibers, ridges, grooves, and pores), geometry describes the larger‐scale shape, curvature, and spatial architecture of a construct. These physical cues are abundant in the native ECM, where they provide essential guidance for cell alignment, migration, and organization [13, 39]. By leveraging topographical and geometrical cues, next‐generation scaffolds can direct in situ tissue regeneration without the need for extensive biochemical supplementation. This approach aligns with the principles of mechanobiology, where physical microenvironmental signals are harnessed to orchestrate cell fate decisions and tissue remodeling, offering a scalable and clinically relevant pathway for regenerative medicine (Details are provided in Section 4.2.4)

### 2.2. Mechanosensing, Mechanotransduction, and Mechanoresponsive Pathways

Mechanosensing is the process by which cells perceive and respond to mechanical cues from their surrounding microenvironment. It represents the first critical step in the broader process of mechanotransduction, where mechanical stimuli are converted into biochemical signals that govern cell behavior and function [13, 40]. In the context of in situ tissue engineering, understanding mechanosensing is pivotal because the success of biomaterial scaffolds hinges not only on their biocompatibility and biodegradability but also on their ability to mimic the mechanical landscape of native tissues and guide cell fate accordingly [9, 11].

As noted already, cells are inherently mechanosensitive. They constantly monitor changes in substrate stiffness, topography, strain, and fluid shear stress through specialized mechanoreceptors, including integrins, ion channels, G protein–coupled receptors, primary cilia, and components of the glycocalyx [25, 41]. These mechanoreceptors interact closely with the cytoskeleton, which acts as a conduit for transmitting mechanical signals deep into the nucleus, ultimately influencing gene expression [42].

One of the central players in mechanosensing is the integrin family of transmembrane receptors. Integrins form FA complexes by binding to ECM ligands and connecting intracellularly to the actin cytoskeleton via proteins, such as talin, paxillin, and vinculin [43, 44]. These complexes function as dynamic hubs for bidirectional signal transduction. They allow cells to pull on the ECM and gauge its rigidity and simultaneously relay external mechanical forces into the cell interior. The stability and maturation of FAs are highly sensitive to substrate stiffness and ligand density, thereby enabling cells to distinguish between soft and rigid environments [44].

Among the primary mechanosensors are mechanosensitive ion channels, such as Piezo‐type mechanosensitive ion channel components 1 and 2 (PIEZO1/2) and transient receptor potential vanilloid 4 (TRPV4). These channels respond to membrane stretch, compression, and osmotic stress by mediating calcium influx, which activates downstream signaling cascades that regulate cell migration, proliferation, and differentiation [45, 46]. In osteogenic lineages, PIEZO‐ and TRPV4‐mediated calcium signaling promotes the expression of osteogenic markers, driving bone regeneration and mineralization [47]. These channels also play key roles in chondrocytes, endothelial cells, and fibroblasts, modulating ECM production and cellular remodeling in response to mechanical forces.

In addition to integrins and ion channels, primary cilia, solitary microtubule‐based organelles protruding from the cell surface, serve as specialized mechanosensory organelles, particularly in chondrocytes and osteocytes. Ciliary bending in response to fluid flow or compression activates Hedgehog, Wnt, and other mechanosensitive signaling pathways, influencing cartilage maintenance and bone homeostasis [48]. Furthermore, actin cytoskeleton not only provides structural integrity but also operates as a dynamic mechanosensor. Through actomyosin contractility, cells apply traction forces on their substrates, probing mechanical properties, such as stiffness and topography. This feedback is coordinated by Rho GTPase signaling and nonmuscle myosin II, modulating cell morphology, FA assembly, and downstream signaling [49]. However, scaffold stiffness that deviates from physiological norms can lead to aberrant cytoskeletal contractility, impairing tissue formation and regeneration. Additionally, the transcriptional regulators, YAP and TAZ, act as nuclear effectors of mechanical signaling. On stiff substrates, YAP/TAZ translocate to the nucleus, promoting proliferation and osteogenic differentiation. Conversely, on soft matrices, YAP/TAZ remain cytoplasmic and inactive, favoring adipogenic or chondrogenic differentiation [10]. This stiffness‐dependent regulation is critical for directing lineage specification in scaffold‐based tissue regeneration.

Also, tailoring the mechanical properties of biodegradable scaffolds is essential to elicit appropriate mechanosensory responses. For example, bone regeneration requires scaffolds with an elastic modulus of ∼10–30 GPa to promote osteogenesis, whereas cartilage repair benefits from softer scaffolds (∼0.1–2 MPa) that support chondrogenesis [12]. Finally, incorporating nanoscale topographical cues, such as grooves, ridges, and pits, further enhances mechanotransduction by influencing cytoskeletal alignment, FA formation, and gene expression, ultimately guiding cell migration, differentiation, and tissue architecture [50].

### 2.3. Mechanotransduction

**FIGURE 1:**
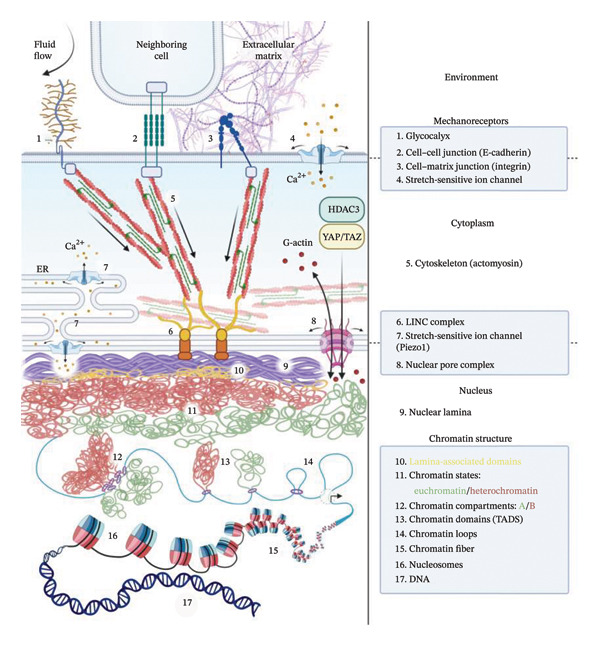
Overview of mechanotransduction pathways. Mechanical cues are detected by mechanoreceptors, such as integrins, mechanosensitive ion channels, primary cilia, and the glycocalyx (#1–4), which transmit forces to the actomyosin cytoskeleton (#5) or trigger calcium influx. Cytoskeletal contractions relay forces to the LINC complex (#6) at the nuclear envelope, deforming it and activating stretch‐sensitive ion channels (#7) for further calcium signaling. This mechanical input dilates nuclear pores (#8), enhancing nuclear import of YAP/TAZ and other transcription factors, while also modulating actin and HDAC3 trafficking. Forces transmitted via the LINC complex reach the nuclear lamina (#9) and chromatin (#10–17), influencing chromatin architecture and gene expression. Adapted with permission from [51].

Mechanotransduction is the fundamental biological process through which cells convert mechanical stimuli from their environment into biochemical signals that regulate gene expression, cell behavior, and tissue development. As shown in Figure 1, at the core of mechanotransduction is a cascade of events that begins with mechanosensing mechanical cues through receptors, such as integrins, mechanosensitive ion channels, primary cilia, and cell surface glycocalyx components. These sensors transmit physical signals to the cell interior via the cytoskeleton, which acts as both a structural support and signaling highway [25, 40, 51].

Several distinct but interconnected mechanisms contribute to mechanotransduction, including FAs, cytoskeletal force transmission, ion channel activation, and nuclear signaling. The following subsections briefly discuss these pathways and implications for scaffold‐guided regeneration.

#### 2.3.1. The Cytoskeleton and Force Transmission

The cytoskeleton serves as the principal internal architecture of the cell, composed primarily of actin filaments, microtubules, and intermediate filaments. It not only provides structural support but also plays a central role in mechanotransduction, the conversion of mechanical signals into biochemical responses [52]. Notably, cells sense external forces through integrin‐based FAs, which physically link the ECM to the cytoskeleton via transmembrane receptors and adaptor proteins, such as talin, paxillin, and vinculin.

When mechanical cues, such as tension, shear, or compression, are applied, they are transmitted across the plasma membrane and propagated through the cytoskeletal network. Actomyosin contractility, mediated by nonmuscle myosin II, enables cells to actively probe and respond to matrix stiffness, while stress fibers reorganize in response to directional strain. These mechanical inputs modulate nuclear shape and influence the localization and activity of transcriptional regulators, such as YAP/TAZ, which are sensitive to cytoskeletal tension and substrate rigidity [53].

Importantly, this force transmission pathway is bidirectional: That is, cells not only sense and respond to mechanical cues but also exert forces back on their environment, remodeling the ECM and altering the mechanical niche. In the context of in situ tissue engineering, tuning scaffold properties to engage the cytoskeletal machinery appropriately is essential for guiding cell fate decisions, maintaining tissue organization, and promoting regenerative outcomes [54].

#### 2.3.2. FA‐Mediated Signaling

One of the primary pathways of mechanotransduction involves FAs, which are multiprotein complexes linking the ECM to the intracellular actin cytoskeleton. Integrins, when activated by ECM binding, cluster and recruit adaptor proteins, such as talin, paxillin, and vinculin, forming mechanosensitive hubs that link the ECM to the actin cytoskeleton [43]. These FAs initiate signaling through focal adhesion kinase (FAK), which in turn engages downstream mitogen‐activated protein kinase (MAPK) and phosphoinositide 3‐kinase/protein kinase B (PI3K/Akt) pathways to regulate cytoskeletal dynamics, cell survival, migration, and differentiation [55]. Substrate stiffness profoundly affects FA dynamics. On stiffer scaffolds, increased integrin engagement and cytoskeletal tension lead to more robust FA formation and enhanced activation of downstream signaling, driving osteogenic differentiation in mesenchymal stem cells (MSCs) [9, 56].

#### 2.3.3. Cytoskeletal Tension and the Nucleus

The actin cytoskeleton, in conjunction with nonmuscle myosin II, generates contractile forces that allow cells to probe their physical environment. These contractile forces are transmitted to the nucleus via the linker of nucleoskeleton and cytoskeleton (LINC) complex, altering nuclear shape and chromatin organization. This mechanical coupling can directly influence the activity of nuclear pores and the accessibility of transcription factors to DNA [57]. As noted earlier, mechanical signals also modulate the localization and activity of transcriptional coactivators, such as YAP and TAZ. On stiff substrates or under high tension, YAP/TAZ translocate to the nucleus to promote the expression of genes associated with cell proliferation and osteogenesis. However, on soft substrates, YAP/TAZ remain cytoplasmic and inactive, favoring adipogenesis or quiescence [10].

#### 2.3.4. Ion Channel Activation

Mechanosensitive ion channels are pivotal regulators of cellular mechanotransduction, acting as direct converters of mechanical stimuli into biochemical signals. Key channels, such as PIEZO1/2, TRPV4, and TREK‐1, respond to membrane stretch, pressure, and fluid flow by mediating ion fluxes, primarily of calcium (Ca^2+^). Upon activation, these ion channels initiate intracellular cascades that control various aspects of cell fate and tissue remodeling. The influx of calcium serves as a secondary messenger that activates: (a) MAPK pathways, to promote proliferation and differentiation; (b) calcineurin–NFAT signaling, to regulate gene transcription and matrix synthesis; and (c) calmodulin‐dependent kinases (CaMKs), influencing cytoskeletal reorganization and mechanoadaptation [46, 47, 58].

In osteoblasts, mechanosensitive ion channel activation enhances mineralization, while in chondrocytes, it stimulates ECM production, supporting bone and cartilage regeneration. These processes are essential for maintaining tissue homeostasis and are critical targets in mechanobiology‐mediated tissue engineering strategies.

#### 2.3.5. Mechanotransduction in Scaffold‐Guided Regeneration

In the context of biodegradable scaffolds, mechanotransduction is a central mechanism by which cells interpret scaffold‐derived mechanical cues. For instance, scaffolds designed with bone‐like stiffness (∼10–30 GPa) facilitate YAP nuclear localization and FA maturation, promoting osteogenesis. Conversely, scaffolds mimicking cartilage (∼0.1–2 MPa) stimulate pathways associated with chondrogenesis, such as SOX9 activation and Wnt modulation [12].

Additionally, fabricating dynamic or “smart” scaffolds that evolve in stiffness or degrade under mechanical load offers additional mechanotransductive cues that mirror the natural remodeling of tissues [59]. Furthermore, the incorporation of nanotopographic features, such as aligned fibers or patterned grooves, enhances mechanotransductive signaling by directing cytoskeletal alignment and guiding stem cell fate [13, 50].

Overall, by integrating mechanically tuned scaffolds with controlled degradation profiles and surface architecture, it could actively steer cellular mechanotransduction pathways. This approach could enable precise control of tissue formation in situ, aligning scaffold design with biomechanical and regenerative needs.

#### 2.3.6. Dysregulated Mechanotransduction

When mechanotransduction is impaired—either through scaffold designs that are too stiff, too soft, or lacking appropriate topographical cues—cells may exhibit abnormal contractility, nuclear distortion, and dysfunctional gene expression [49]. This can lead to poor matrix deposition, inflammation, or tissue fibrosis, ultimately compromising scaffold integration and regeneration.

### 2.4. ECM Remodeling and Feedback Loops

As expected, cells are not passive recipients of mechanical signals. Through traction forces transmitted via integrin‐based adhesions and cytoskeletal contractility, they actively reshape their ECM. This remodeling, comprising fiber alignment, stiffening, and degradation, creates a dynamic bidirectional feedback loop (cells mechanically alter their microenvironment, which in turn modifies cell behavior) [60, 61].

In 3D settings, the ECM exhibits complex behaviors, such as nonlinear elasticity, viscoelastic stress relaxation, and plastic deformation (all of which regulate how cells apply forces and respond over time) [62]. As cells exert traction on their surroundings, they induce the alignment and stiffening of ECM fibers along the direction of applied forces. Remarkably, this mechanical remodeling can extend the region of increased stiffness to zones up to 10 times larger than the cell itself, influencing not only individual cell behavior but also enabling long‐range mechanical communication across cell populations [63].

This ECM–cell feedback loop plays a crucial role in development, wound healing, and disease progression. For instance, during fibrosis or tumor progression, excessive ECM stiffening sustains a positive feedback loop that perpetuates pathological cell behavior [64, 65].

In tissue engineering, harnessing this concept allows scaffolds to guide self‐organization. Additionally, biomaterials may be designed to be remodeled by cell‐driven forces, which then reinforce or alter the scaffold’s mechanics to direct differentiation, migration, and tissue maturation in situ [15]. By integrating feedback‐responsive biomaterials, engineered constructs can mimic the natural process of tissue morphogenesis and functional repair.

## 3. In Situ Tissue Engineering: The New Paradigm in Tissue Engineering

Despite considerable progress, tissue engineering continues to face major challenges, including architectural complexity, insufficient vascularization, immune rejection, and stringent regulatory constraints. Contrary to ex vivo or conventional tissue engineering, where functional tissues are fabricated outside the body using cells, scaffolds, and bioactive factors before implantation, in situ tissue engineering adopts a fundamentally different approach by harnessing the body’s intrinsic regenerative potential and biomechanical environment to restore injured or dysfunctional tissues [66, 67].

Conventional tissue engineering typically involves in vitro cell expansion, scaffold seeding, and prolonged bioreactor culture before implantation. Although effective under controlled laboratory conditions, these processes are costly, labor‐intensive, and dependent on specialized infrastructure and expertise. Moreover, the use of exogenous cells and biomaterials can elicit immune responses, increasing the risk of graft rejection and implant failure. Following implantation, engineered constructs frequently experience poor vascularization, leading to ischemia, nutrient deprivation, and impaired function, particularly in large or metabolically active tissues (bone, cardiac muscle, skeletal muscle, etc.) [3, 5]. Scalability further limits clinical translation, as manufacturing clinical‐grade, cell‐based constructs while maintaining quality assurance is resource‐intensive and time‐consuming, compounded by stringent regulatory processes [4, 65]. These persistent obstacles have motivated the shift toward in situ tissue engineering, a paradigm offering reduced complexity, enhanced host integration, and improved translational feasibility.

In principle, in situ tissue engineering activates endogenous repair mechanisms by delivering bioactive or instructive scaffolds directly to the defect site, enabling regeneration within the native physiological and mechanical milieu. This approach promotes seamless integration with host tissues, yielding regenerated structures that closely replicate native composition, architecture, and function. By minimizing reliance on donor‐derived cells, in situ strategies reduce immune and contamination risks while improving safety and reproducibility. Clinically, in situ tissue engineering provides simplified and adaptable workflows compatible with minimally invasive interventions and personalized medicine, leveraging each patient’s intrinsic cells, immune responses, and biomechanical cues for efficient, targeted regeneration [6, 68].

Mechanobiology offers a conceptual framework for the rational design of scaffolds that actively engage resident cells through mechanical signaling. By recapitulating or amplifying native biomechanical stimuli, such scaffolds can recruit, align, and direct cell differentiation while promoting in situ ECM deposition [15, 69]. Mechanically adaptive materials further enhance regeneration by dynamically responding to local physiological stresses, reinforcing repair through feedback mechanisms, such as tension‐mediated ECM remodeling, without dependence on exogenous growth factors [70, 71]. This intrinsic adaptability is particularly advantageous for complex, load‐bearing tissues, such as bone, cartilage, and tendon, where precise mechanical fidelity is critical for restoring structural and functional integrity. However, the full potential of mechanobiology‐mediated in situ tissue engineering would likely be achieved presumably after addressing some translational challenges (details in Section 6).

## 4. ECM Mimetic Bioactive Scaffold Designs

Advances in biomaterials, molecular biology, and biofabrication have enabled the design of bioactive 3D multifunctional scaffolding systems that mimic the natural ECM, providing an impetus to our quest for translational tissue engineering. As expected, an ideal 3D scaffold ought to mimic the ECM by providing cells with both structural support and transport properties, as well as metabolic cues essential for adhesion, migration, proliferation, differentiation, and ultimately guiding the development of complex tissue architectures. In this regard, a multifunctional scaffold must incorporate biological and chemical cues (derived from ECM, encapsulated with bioactive molecules, such as growth factors, small molecules, and short peptides), and mechanical or topographical cues from stiffness, surface topography, and geometry, among others. Details are provided herein.

### 4.1. Bioactive Biomaterial Scaffolds

Biomaterial scaffolds are the 3D support for seeding the cells. They are naturally or synthetically made. Natural biomaterials, such as collagen, alginate, and fibrin, have demonstrated excellent biocompatibility, offering bioactive surfaces that closely mimic native ECM components and support cellular function [72]. However, these natural polymers often exhibit limitations in terms of poor mechanical strength and degradation control. To overcome these challenges, synthetic polymers, such as polylactic acid (PLA), polyglycolic acid (PGA), and polycaprolactone (PCL), have been employed due to their tunable mechanical properties and customizable degradation rates [73]. In conventional tissue engineering, biomaterials are seeded with cells ex vivo. Central to this strategy is the tissue engineering triad, which comprises the following: (a) cells, which serve as the primary biological units for tissue formation; (b) scaffolds, the framework for cell attachment, proliferation, and ECM deposition; and (c) signaling cues (including growth factors, small molecules, and mechanical stimuli), which guide cellular processes, such as differentiation, angiogenesis, and tissue maturation [74]. Contrary to conventional ex vivo tissue engineering, in situ tissue engineering stimulates the body’s own endogenous cells (within the microenvironment) to the targeted injury sites for tissue regeneration. By incorporating bioactive molecules (small molecules, chemokines, and growth factors) in the 3D biomaterial scaffolds, endogenous cells are recruited to regulate tissue repair and neovascularization, among others. Therefore, to achieve this, modern delivery systems are engineered to mimic the body’s natural healing environments, ensuring bioactive molecules are released at precise locations and times [75].

### 4.2. Bioactive Molecule Delivery Strategies

Key bioactive molecules, which drive tissue repair and regenerative processes (angiogenesis, osteogenesis, and stem cell recruitment), include vascular endothelial growth factor (VEGF), bone morphogenetic protein‐2 (BMP‐2), stromal cell‐derived factor‐1 alpha (SDF‐1α), substance P (SP), chemokines, and proinflammatory cytokines. However, the therapeutic potential of these bioactive molecules relies on developing optimized delivery methods. Thus, strategies, such as sustained release, load‐responsive scaffolds, supramolecular encapsulation, and stimuli‐responsive systems, are being explored to enhance bioactivity retention, cellular uptake, and localized delivery [76, 77]. A brief description of some promising delivery systems is further discussed.

#### 4.2.1. Sustained and Control Release of Bioactive Molecules

A central principle of in situ tissue engineering is the localized and sustained delivery of bioactive molecules to the injury site. Sustained delivery systems are designed to maintain therapeutic concentrations of growth factors within the target tissue microenvironment, thereby enhancing tissue regeneration while minimizing systemic exposure and associated adverse effects. Among the most employed growth factors are VEGF, BMP‐2, and SDF‐1*α*, each playing a distinct yet synergistic role in the regenerative cascade [78].

Specifically, VEGF is a potent angiogenic factor that promotes endothelial cell proliferation, migration, and neovascularization, which are essential for restoring vascular networks in injured tissues. BMP‐2, a member of the transforming growth factor‐beta (TGF‐β) superfamily, induces osteogenic differentiation of MSCs, thereby driving the formation and mineralization of new bone tissue. Conversely, SDF‐1*α* primarily functions as a chemoattractant, recruiting progenitor and stem cells to the defect site while simultaneously facilitating tissue remodeling and repair. The coordinated release of these molecules in a spatiotemporal sequence is therefore critical for orchestrating complex regenerative processes [78].

The release kinetics of these bioactive molecules are determined by their mode of encapsulation within 3D biomaterial scaffolds. These scaffolds are engineered to mimic the structural and functional attributes of the native ECM, thereby serving as both a physical support for cellular attachment and a dynamic reservoir for biochemical signaling. Controlled release is achieved through precise modulation of scaffold composition, porosity, degradation rate, and affinity for the encapsulated molecules. This approach allows for the sequential and sustained release of growth factors, closely replicating the physiological healing cascade [78].

For instance, Docheva and Ryu [79, 80] developed a dual‐delivery system in which scaffolds were sequentially loaded with VEGF and BMP‐2. VEGF was released during the early phase of healing to stimulate rapid angiogenesis, while BMP‐2 was gradually released at later stages to enhance vascular stabilization, osteogenic differentiation, and bone mineralization. This temporally controlled release pattern resulted in superior tissue integration and more effective replication of natural bone healing compared to single‐factor delivery systems.

Building upon these concepts, environmentally responsive or “smart” scaffolds have emerged as an advanced strategy for in situ tissue engineering. Such scaffolds are designed to release their therapeutic cargo in response to specific physiological stimuli within the injury microenvironment, such as pH changes, enzymatic activity, oxidative stress, or mechanical loading. It has been observed that, chitosan‐based hydrogels/nanoparticles composite engineered with different functional groups created a dynamic 3D scaffold capable of controlled, on‐demand release of encapsulated bioactive factors [81]. This responsive behavior enables precise spatiotemporal regulation of growth factor availability, allowing for synchronization of biochemical signaling with the distinct stages of tissue repair.

Overall, these strategies highlight the importance of sustained and stimuli‐responsive release systems in modern in situ tissue engineering. By integrating bioactive molecules within ECM‐mimicking scaffolds and controlling their delivery profiles, it is possible to direct cellular recruitment, angiogenesis, and differentiation in a highly coordinated manner, thereby accelerating functional tissue regeneration while reducing the risks associated with uncontrolled or systemic growth factor administration.

#### 4.2.2. Mechanically Triggered Release Systems

Mechanical forces are fundamental regulators of tissue homeostasis and regeneration, particularly in load‐bearing tissues, such as bone, tendon, and cartilage. Beyond providing structural integrity, these forces govern cellular behavior through mechanotransduction (a process by which physical stimuli are converted into biochemical signals) that direct cell proliferation, migration, differentiation, and ECM remodeling [82]. In the context of in situ tissue engineering, leveraging these endogenous mechanical cues provides an opportunity to design smart, stimuli‐responsive delivery systems that release therapeutic agents in synchrony with physiological loading patterns.

Mechanically responsive release systems are typically constructed from dynamic biomaterials, such as hydrogels, elastomers, and polymer composites, which undergo structural deformation when subjected to mechanical stress. This deformation can trigger the release of encapsulated bioactive molecules through several mechanisms, including (a) scaffold pore expansion, where there is transient increase in pore size to facilitate the diffusion of entrapped molecules; (b) scaffold degradation is induced, leading to breakdown of crosslinked networks; and (c) bond rupture, where mechanical cleavage of labile chemical bonds, results in release of tethered factors [83, 84].

An illustrative example is the use of RGD peptide‐functionalized hydrogels, which promote *integrin-mediated cell adhesion* and enhance *cellular mechanosensitivity*. These functionalized scaffolds improve growth factor retention while simultaneously fostering mechano‐responsive signaling within the microenvironment [81, 85]. This dual function allows the scaffold to act not only as a structural support and reservoir but also as a bioactive interface that interacts dynamically with resident cells.

A critical advantage of mechanically triggered systems is their ability to replicate the temporal sequence of natural tissue repair processes. For example, during the early stages of tissue loading, scaffolds can be engineered to release angiogenic and chemotactic factors, such as VEGF and SDF‐1. These molecules facilitate vascular ingrowth and progenitor cell recruitment, which are essential for establishing a well‐perfused, regenerative niche. As tissue repair progresses and mechanical loading patterns evolve, the scaffold can subsequently release osteogenic factors, such as BMP‐2, to promote osteogenic differentiation, mineralization, and matrix maturation [86, 87].

By integrating mechanobiology with controlled release, these systems enable spatiotemporal regulation of growth factor availability that mirrors the body’s natural healing cascade. This approach represents a paradigm shift from passive scaffolding toward active, mechanically interactive biomaterials capable of sensing their environment and delivering therapeutic cues in real‐time. Such technologies hold significant promise for regenerating load‐bearing tissues where mechanical forces play a pivotal role in both tissue damage and repair.

#### 4.2.3. Sequential Release Systems

Tissue regeneration is inherently governed by a highly coordinated spatiotemporal sequence of biological signals, in which the timing, concentration, and localization of biochemical cues are tightly regulated. During natural healing processes, distinct signaling molecules are released in a defined order to orchestrate cell recruitment, vascularization, matrix deposition, and tissue remodeling. A classic example is observed in bone repair, where angiogenesis precedes osteogenesis. In the early phase of healing, VEGF is upregulated to stimulate endothelial cell migration and neovascularization, establishing a vascular network that provides oxygen and nutrients to the regenerating tissue. Once vascularization is established, BMP‐2 is subsequently expressed to promote osteogenic differentiation and bone mineralization, thereby completing the regeneration process [81].

Replicating this natural sequence of events in engineered systems is essential for achieving functional tissue regeneration. To address this challenge, sequential delivery platforms have been developed to spatially and temporally regulate the release of bioactive molecules (Table 1) [78, 81]. These systems are designed to mimic the body’s intrinsic healing cascades by providing localized, phase‐specific cues that direct cellular behavior at each stage of repair.

**TABLE 1:** Overview of sequential delivery strategies.

Strategy	Representative agents	Mechanism/key feature	Advantages	Limitations	References
Sustained release systems	VEGF, BMP‐2, and SDF‐1	Encapsulation in hydrogels, scaffolds, or nanoparticles to maintain release over time	Provides constant therapeutic levels and reduces systemic side effects	Risk of initial burst release; limited fine‐tuning of kinetics	[78, 81]
Mechanically responsive systems	BMP‐2, SDF‐1, and VEGF	Smart polymers or hydrogels that discharge bioactive in response to load or stress	Aligns delivery with natural tissue loading, useful in bone and tendon repair	Fabrication complexity; variable response in vivo	[75, 79]
Supramolecular carriers and nanoparticles	VEGF, insulin, lysozyme, and chemokines	Noncovalent assemblies or functionalized nanoparticles that protect and release proteins	Preserves bioactivity, allows intracellular targeting, and avoids burst release	Requires functionalization; stability can be context‐dependent	[88]
Gradient and sequential release systems	VEGF, BMP‐2, and SDF‐1	Layered scaffolds or compartmentalized hydrogels programmed for staged release	Reproduces natural healing cascades (angiogenesis before osteogenesis)	Technically demanding; limited large‐scale translation	[79, 81]
Stimuli‐responsive matrices	SDF‐1, BMP‐2, and VEGF	Release triggered by changes in pH, enzyme activity, or redox environment	Enables site‐specific, adaptive delivery at the wound microenvironment	Potential off‐target activation; requires careful material design	[80, 89]

For instance, advanced biomaterials, such as multilayered hydrogels and dual‐compartment nanoparticles, allow precise modulation of both timing and location of growth factor release. Early‐release compartments deliver angiogenic factors, such as VEGF, to initiate vascular network formation, while subsequent layers or compartments gradually release osteogenic or regenerative factors, such as BMP‐2, to stimulate matrix deposition and mineralization. This strategy closely mimics the temporal hierarchy of natural healing, promoting orderly tissue development and preventing premature or uncoordinated signaling. Docheva et al. [79] and Leijten et al. [90] demonstrated that scaffolds with a VEGF‐first/BMP‐2‐later release pattern achieved superior vascularization and bone formation compared to single‐factor delivery systems.

In related studies, stimuli‐responsive scaffolds were fabricated to mimic the dynamic microenvironment for wound healing. In this work, chitosan‐based matrix scaffolds were engineered to respond to pH changes, redox conditions, or specific enzymes, releasing growth factors only when triggered by the local environment [80, 91]. This adaptability enables phase‐specific release of bioactive factors that aligns with the natural progression of wound healing.

Emerging delivery systems combine spatiotemporal biochemical control with mechanical responsiveness, creating fully dynamic scaffolds capable of adapting to both biological signals and physical forces. Examples include the following: (a) load‐responsive scaffolds that alter release profiles in response to tissue loading and mechanical stress; (b) supramolecular nanoparticles that self‐assemble or disassemble under specific conditions to deliver chemokines or growth factors; and (c) stimuli‐sensitive hydrogels that integrate biochemical, mechanical, and environmental triggers into a single platform [83, 88, 92]. Table 1 provides a summary of delivery strategies for small molecules, chemokines, and growth factors.

Overall, the integration of spatiotemporally controlled delivery systems for small molecules, chemokines, and growth factors is central to the advancement of mechanobiology‐mediated in situ tissue engineering. Cutting‐edge platforms, including load‐responsive scaffolds, supramolecular nanoparticles, and stimuli‐sensitive hydrogels, allow for the precise control of regenerative cues. These biomimetic delivery systems enable therapeutic interventions that closely mirror the body’s natural healing cascades, representing a transformative step toward functional tissue regeneration [83, 88, 92].

#### 4.2.4. Topography‐Mediated Biomaterials

Many studies on topography use 2D substrates or thin films, which mainly reveal how cells respond to surface‐level contact guidance cues, such as alignment, elongation, migration, and neurite extension [84, 93–95]. However, successful in situ tissue engineering depends largely on 3D architecture, where pore size, interconnectivity, and permeability regulate cell infiltration, mass transport, and vascular ingrowth [93, 96–100]. Because 2D and 3D systems influence regeneration through different mechanisms, we briefly distinguish them here for clarity. The influence of topographical features on cell behavior has been recognized for over a century [101]. The concept of contact guidance, describing the alignment of cells along surface topographies, was formally introduced in the 1950s [102]. Building on this, Curtis and Varde (1964) demonstrated that fibroblasts align parallel to silica fibers with diameters ranging from 10 to 30 μm. Thereafter, a growing body of evidence has shown that microscale and nanoscale surface features can guide cell alignment, elongation, migration, and differentiation [103–105]. Topographical cues, such as grooved substrates and aligned fiber, mimic aspects of the native tissues that play a crucial role in guiding cell behavior. These topographical designs are effective in enhancing neural and musculoskeletal regeneration.

**FIGURE 2:**
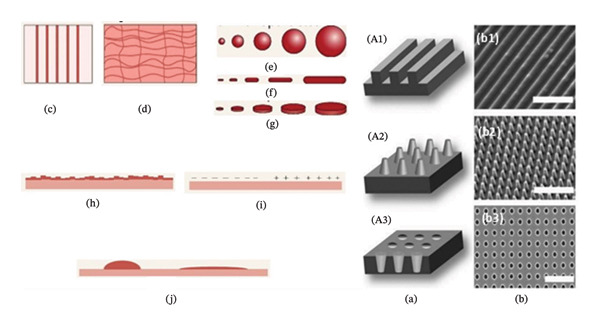
Schematic illustrations: (a) (A1–A3) and corresponding SEM images (B1–B3) of representative nanotopographical surface geometries: nanografting (A1, B1: scale bar 5 µm), nanopost (A2, B2: scale 5 µm), and nanopit array (A3, B3: scale bar 1 µm). Adapted with permission from [108]. (b–j) Surface properties of biomaterials—including topographical guidance, nanoscale shape and dimensions, surface roughness, charge, and wettability: alignment guidance (c), pattern guidance (d), 0D particle shapes (e), 1D particle size (f), 2D particle size (g), roughness (h), charge (i), and wettability (j). Adapted with permission from [109].

As noted above (Section 2.2), cells interact with topographical features through integrin receptors, which cluster at surface contact points to form FA‐specialized mechanosensory complexes that anchor the cytoskeleton to the substrate. These FAs initiate mechanotransduction, converting mechanical input into biochemical signals that regulate cytoskeletal organization, nuclear shape, and gene expression [84, 106, 107]. The nature of this interaction is influenced not only by the geometry but also by the topographical pattern (such as grooves, ridges, pits, roughness, charge, wettability, and stiffness; Figure 2) to impact cellular behavior by guiding alignment, elongation, and differentiation (Table 2).

**TABLE 2:** Summary of topography‐mediated biomaterials.

Topography	Material	Cell type	Observed response	Fabrication method	Application domain	References
Nanogrooves (500–1050 nm)	PDMS	hBM‐MSCs	↑Adhesion, ↑intracellular stiffness, stemness preserved	Plasma treatment + uniaxial strain	Stem cell mechanobiology	[84]
Aligned nanofibers (∼500 nm)	PLLA/PCL	hBM‐MSCs	↑Elongation, ↑SCX/COL1 expression, tenogenic differentiation	Electrospinning with rotation collector	Tendon tissue engineering	[96]
Random nanofibers	PLLA/PCL	hBM‐MSCs	↑Osteogenic markers (Runx2, osteopontin)	Electrospinning (no alignment control)	Bone tissue engineering	[96]
Microgrooves (10–20 µm)	PLA	Schwann cells	Pronounced alignment along groove	Microfabrication	Neural guidance	[96]
Nanofibers (283 nm)	PLA	Neural stem cells (NSCs)	↑Oligodendrocyte differentiation (+40%)	Electrospinning	Neural lineage bias	[94]
Nanofibers (749 nm)	PLA	NSCs	↑Neuronal differentiation (+20%)	Electrospinning	Neuronal fate	[94]
Nanopatterned ridges	Glass (no ECM coating)	PC12 neuron‐like cells	↑Neurite outgrowth (+200%)	Reactive ion etching	Neurogenesis without ECM	[94]
Aligned microfibers	PLGA	NSCs	↑Elongation ↑ NSC maturation	Microfluidic fiber spinning	Neural scaffold design	[99]
Microgrooves	Gelatin–genipin	Fibroblasts	↑Elongation ↑alignment ↑Directional migration	Soft lithography	Wound healing, dermal regeneration	[93]
Geometric micropattern	PDMS	hBM‐MSCs	Osteogenic vs. adipogenic fate based on shape (concave vs. circular)	Microcontact printing	Shape‐guided stem cell differentiation	[96]
Nanonodules (300–560 nm)	Titanium/TiO_2_	hBM‐MSCs	↑ALP ↑collagen ↑osseointegration (in vivo)	Acid etching/surface oxidation	Bone regeneration	[100]

These features mimic the structural anisotropy of the native ECM, activating integrin‐mediated contact guidance and downstream mechanotransduction signaling. Notably, such physical cues can direct cell fate decisions even in the absence of soluble biochemical inducers. Mahdavian et al. [95] fabricated nanogroove (500–1050 nm depth) pattern on polydimethylsiloxane (PDMS) substrates via plasma treatment under uniaxial mechanical strain. When cultured on these substrates, human bone marrow–derived mesenchymal stem cells (hBM‐MSCs) exhibited enhanced adhesion, increased intracellular stiffness, and improved maintenance of stemness compared to flat controls [95]. Similarly, aligned nanofibrous (diameters ∼ 500 nm) scaffolds composed of poly(L‐lactic acid) (PLLA) and PCL successfully directed hBM‐MSCs toward a tenogenic lineage [95]. These scaffolds promoted cell elongation, nuclear alignment, and upregulation of tenogenic markers, such as scleraxis and collagen type I, with in vivo formation of tendon‐like tissue [95]. In contrast, randomly oriented fibers induced osteogenic differentiation [95], highlighting how spatial alignment controls stem cell fate. In neural tissue models, Liu et al. [94] demonstrated that Schwann cells cultured on microgrooved surfaces (groove width: 10–20 µm; depth: 2–5 μm) exhibited strong alignment along the groove axis, matching the natural width of the cells. Furthermore, neural stem cells (NSCs) cultured on electrospun nanofibers (diameter 238 nm) displayed enhanced oligodendrocyte differentiation by 40%, while 749 nm fibers favored neuronal differentiation by 20% [94]. In parallel, PC12 neuron‐like cells grown on nanopatterned glass substrates (fabricated by reactive ion etching) showed a 200% increase in neurite outgrowth compared to flat controls [94], indicating that topography alone is sufficient to drive neurogenesis.

Micro‐ and nanotopographical cues are critical regulators of stem cell behavior and tissue regeneration. Microcontact printing of adhesive geometries, such as stars, pentagons, and circles, on PDMS surfaces has shown that cell shape and edge curvature strongly influence lineage commitment [93]. Concave geometries promoted osteogenesis, while rounded shapes favored adipogenesis in hBM‐MSCs [93]. In dermal regeneration, gelatin–genipin microgrooves enhanced fibroblast elongation and guided directional migration [93].

Substrate stiffness also plays a pivotal role in directing stem cell fate. Soft matrices resembling brain tissue (∼0.1–1 kPa) promote neuronal differentiation; intermediate stiffness substrates, such as muscle (∼8–17 kPa), favor myogenesis; and rigid substrates mimicking bone (> 30 kPa) drive osteogenesis. This mechanosensitive behavior occurs through cytoskeletal tension and mechanotransduction pathways, which modulate nuclear shape and gene expression, ultimately guiding lineage specification [97, 98].

For musculoskeletal repair, aligned fibrous scaffolds made of silk fibroin, poly(ester urethane) urea, and fibrinogen promoted tenogenic differentiation, upregulated tendon markers, such as decorin and tenomodulin, and enhanced mechanical properties under dynamic strain [100]. Conversely, randomly oriented mineral‐coated PCL fibers supported osteogenesis by increasing expression of Runx2 and osteopontin. In neural regeneration, an aligned poly(lactic‐co‐glycolic acid) (PLGA) fibers fabricated using a microfluidic system, significantly enhanced neurite outgrowth and NSC maturation [100]. Similarly, nanopatterned PLA fibers releasing L‐lactic acid improved Schwann cell survival and neuronal support [100]. Titanium and titanium dioxide (TiO_2_) surfaces with nanonodules (300–560 nm) further enhanced osteogenic differentiation and bone integration in vivo [100].

Put together, while topography‐mediated biomaterial looks promising, it is important to incorporate 3D dynamic stimuli‐responsive in topography‐mediated biomaterial scaffolds to replicate the native ECM tissue microenvironment and complexity [98]. This is particularly important given that conventional 2D cultures fail to faithfully mimic the 3D architecture of the ECM.

#### 4.2.5. Mechanical Evolution for In Situ Regeneration

A critical determinant of in situ scaffold performance is its ability to provide mechanical support while surrounding tissues gradually remodels to replace the in situ scaffold. Note that cell behavior is strongly regulated by (a) the viscoelastic and time‐dependent properties of the surrounding matrix, and (b) iterative ECM–cell mechanical feedback (during repair); thus, scaffold mechanics should be modeled across the three transition stages (inflammation, proliferation, and remodeling) [49, 59–62].

**FIGURE 3:**
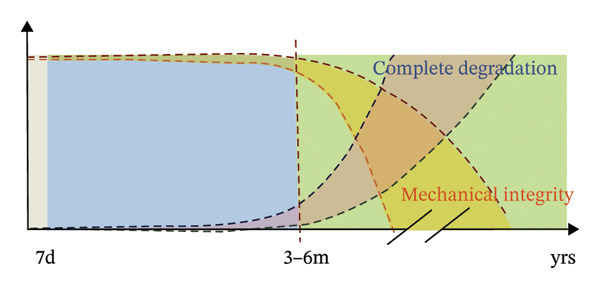
An illustration of bone healing stages, with the gradual degradation of a biodegradable metal implant. Reproduced with permission from [110]. Copyright 2016, Springer Nature.

During the inflammatory phase (days 0–7, Figure 3), the scaffold should retain sufficient stiffness to (a) stabilize the defect, (b) prevent deleterious deformation, and (c) appropriately modulate early immune activity [49, 60]. As expected, excessive early softening undermines structural integrity and subsequently triggers unfavorable inflammation response; it is crucial that the right biomaterials (e.g., PLGA, with favorable degradation rate) are designed (using higher crosslink density, initial crystallinity, network architectures, etc.) to resist rapid modulus loss [60].

It is important to engineer the in situ scaffold (with high porosity and interconnectivity architecture) such that at the proliferative phase (weeks 1–4), the scaffold is able to facilitate cellular infiltration, spreading, neovascularization, and matrix deposition, enabling cells to remodel their niche, while the scaffold provides global support [49, 60–62]. High porosity and interconnectivity can be engineered through (a) hydrolytically and enzymatically degradable linkages, (b) swelling‐responsive hydrogels, and (c) architectures with open [111, 112].

During the remodeling and maturation (weeks–months), the scaffold should gradually soften to permit progressive load transfer to the neo‐tissue while preserving directional mechanical cues (e.g., anisotropy for tendon/nerve) where needed [60].

Put together, controlled degradation rate is a prerequisite (for tailoring scaffold surface roughness, micro‐ and nanotopographic features, among others) for providing mechanical and chemical cues for cell adhesion, spreading, proliferation, and differentiation.

## 5. Tissue‐Specific Advances in In Situ Tissue Engineering

**FIGURE 4:**
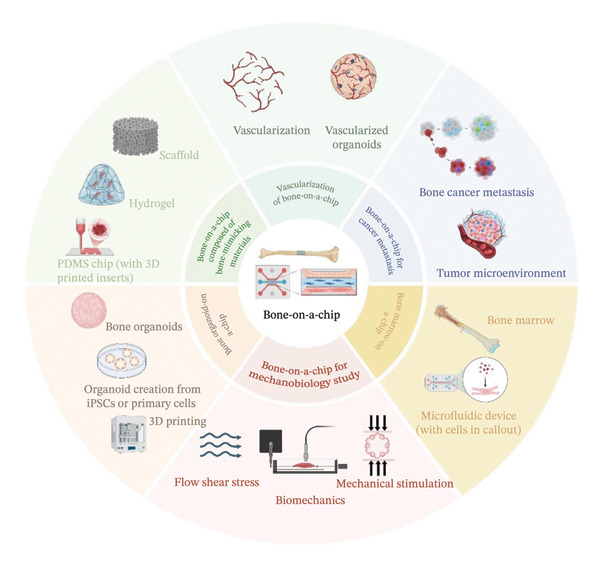
The construction and application of bone‐on‐a‐chip for biomedical applications. Reproduced from [115], Biomedicines, under CC BY 4.0 license.

Mechanobiology‐mediated in situ tissue engineering leverages the body’s natural response to mechanical stimuli to drive tissue regeneration at injury sites. Through mechanotransduction, cells sense and convert mechanical forces into biochemical signals, primarily via integrins, which regulate key functions, such as proliferation, differentiation, and tissue growth [113, 114]. Mechanical cues are fundamental to processes, such as cell division and tissue remodeling [113]. Smart biomaterials, including hydrogels and piezoelectric polymers, are used to fabricate 3D scaffolds that generate bioactive signals. In addition to smart biomaterials, organ‐/tissue‐on‐chip (OoC/ToC) platforms are increasingly used as complementary tools to better replicate dynamic in vivo microenvironments, overcoming limitations of conventional 2D and animal models (Figure 4) [115]. When combined with biomaterial‐based strategies, these systems enable more precise investigation of mechanobiological cues through controlled mechanical stimulation and biomimetic architectures. Together, they enhance scaffold design and validation for improved in situ tissue engineering applications. This section explores applications of this approach in regenerative medicine.

### 5.1. Bone Tissue Regeneration

Hydrogels are emerging as a highly promising smart biomaterial platform for mechanobiology‐mediated bone tissue engineering owing to their tunable mechanical and biochemical properties. These smart biomaterials can be engineered to respond to external stimuli, such as light, heat, pH, and magnetic fields, enabling dynamic modulation of stiffness and functionality [116]. Such adaptability is particularly beneficial for regenerating complex bone‐soft tissue interfaces, where stiffness gradients play a key role in directing cell behavior, promoting osteogenesis, and supporting vascularization.

Hydrogels with optimized stiffness can guide stem cell differentiation and improve bone regeneration outcomes. For instance, in vitro mechanistics have shown that hydrogel with tunable functionalization enhances osteogenic differentiation. In particular, matrices with increased stiffness upregulate osteogenic genes and cytoskeletal tension [117–119]. Furthermore, incorporating osteogenic ions (e.g., Ca^2+^) or BMP‐2 into hydrogen matrix further accelerates mineral deposition and matrix maturation in rodent critical‐size models, showing improved bone filling and vascularization [111, 118].

To improve vascularization, it is possible to incorporate angiogenic molecules, such as deferoxamine, into hydrogel scaffolds to stimulate blood vessel formation, improving bone healing [119]. Additionally, coculturing endothelial cells with MSCs within hydrogels has been shown to promote simultaneous vascularization and overall regenerative outcomes [112].

Beyond small‐animal in vivo study, advanced hydrogen design (sequentially crossed‐linked or dynamic hydrogel) in large‐animal model (e.g., sheep) has supported robust bone formation, improved osseointegration, and mechanical performance [120].

Despite these advances, several challenges remain, including immune rejection, mechanical mismatch between scaffolds and native bone, and incomplete vascularization. To help address these limitations, particularly those arising from conventional 2D culture and animal models, bone‐on‐a‐chip platforms have been developed as complementary tools to better replicate the dynamic in vivo microenvironment.

These systems integrate mechanical stimuli, such as interstitial fluid shear stress and cyclic compression, with biochemical interactions between key cell types, including osteoblasts and osteoclasts, enabling real‐time investigation of bone remodeling processes. Consequently, bone‐on‐a‐chip technologies have supported diverse applications, including vascularized bone models, bone marrow‐on‐chip systems, disease models for metastasis, mechanobiological studies, and the development of bone organoids [115].

### 5.2. Cartilage Repair and OA Management

**FIGURE 5:**
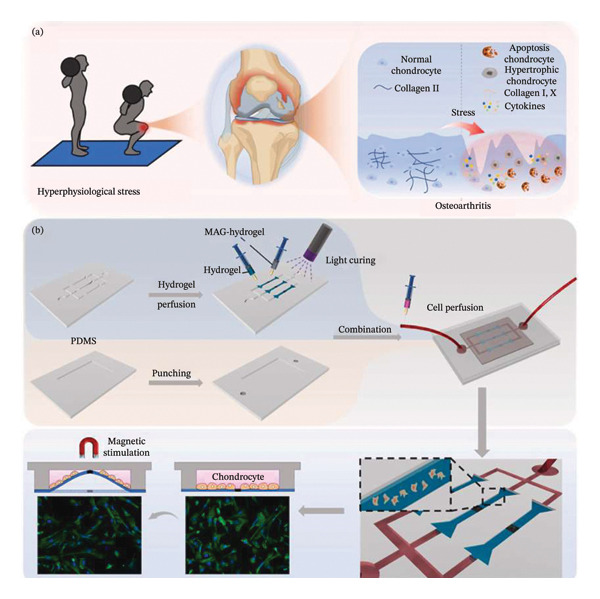
An illustration of cartilage undergoing physiological stress and magnetomechanical loading treatment. Adapted with permission from [122].

OA is a prevalent degenerative joint disease, primarily resulting from cartilage overstrain and mechanical wear, particularly in the aging population [121]. Conventional treatments often fail to restore cartilage integrity, prompting the exploration of mechanobiology‐based approaches. One promising strategy involves magnetomechanical loading, which combines magnetic and mechanical stimuli to modulate cellular behavior and facilitate cartilage repair and regeneration (Figure 5). This technique typically employs the incorporation of magnetic nanoparticles, such as Melanin@Fe_3_O_4_, into stem cells or chondrocytes. When subjected to an external magnetic field, these nanoparticles generate localized mechanical forces that act on both the cells and the surrounding cartilage matrix [122, 123]. The resulting biomechanical stimulation activates critical cellular processes, including chondrogenic gene expression, ECM deposition, and chondrocyte aggregation in in vitro studies [121, 122]. Furthermore, in in vivo, magnetomechanical loading has been shown to reduce OA progression, increase cartilage thickness, and enhance proteoglycan retention in rodent OA models [123].

Beyond promoting matrix synthesis, this method offers several additional advantages, such as (a) enhancement of cell assembly and intercellular communication [124]; (b) stimulation of chondrogenic differentiation; (c) remote control of cellular behavior via magnetic fields [121]; and (d) development of cartilage‐on‐a‐chip platforms for disease modeling and therapeutic testing [122]. However, despite these advancements, further research is needed to optimize magnetic nanoparticle design, refine magnetic field parameters, and evaluate long‐term efficacy and safety in clinical applications.

### 5.3. Cardiac Tissue Regeneration

**FIGURE 6:**
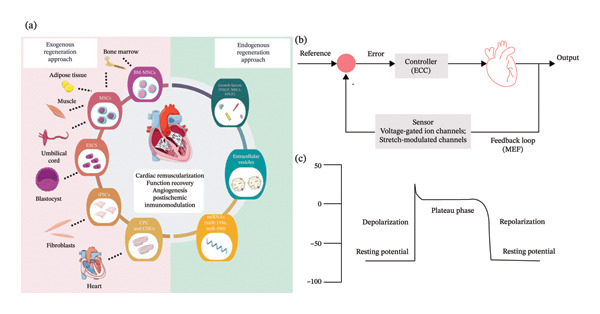
Mechanobiology‐mediated cardiac repair and regulation. (a) Exogenous and endogenous cardiac regeneration strategies. Exogenous approaches rely on the transplantation of stem or progenitor cell–derived constructs (BM‐MNCs, MSCs, ESCs, iPSCs, and CPCs/CDCs), whereas endogenous strategies stimulate the heart’s intrinsic repair capacity through bioactive molecules, growth factors (VEGF, HGF, and bFGF), extracellular vesicles, and regulatory miRNAs (miR‐199a and miR‐590). Both modalities aim to restore cardiac function via angiogenesis, remuscularization, and postischemic immunomodulation. Adapted with permission from [126]. (b) Simplified control–feedback model of the heart’s electromechanical coupling. Electrical excitation triggers contraction (excitation–contraction coupling, ECC), while mechanosensitive ion channels provide feedback (mechanical–electrical feedback, MEF) that regulates action potential and pumping efficiency. (c) Representative phases of the cardiac action potential: resting potential, depolarization, plateau, and repolarization. Adapted with permission from [127].

Myocardial infarction (MI), commonly known as a heart attack, occurs when blood flow to the heart is obstructed, leading to oxygen deprivation and subsequent death of cardiac tissue [125]. Due to the heart’s limited intrinsic capacity to regenerate cardiomyocytes, postinfarction healing often results in scar tissue formation, which impairs cardiac function (Figure 6a). However, recent advances in cardiac tissue engineering have introduced piezoelectric scaffolds as a promising strategy to overcome this limitation. These scaffolds serve as templates for new tissue formation, helping to reduce fibrosis and enhance cardiac repair [126]. Piezoelectric materials, such as polyvinylidene fluoride (PVDF) and its copolymers, can mimic the native electromechanical environment of the myocardium, simultaneously providing mechanical support and electrical stimulation to guide cardiac cell activity [128]. For example, piezoelectric scaffolds enhance cardiomyocyte maturation, electrical coupling, and alignment under electromechanical loading in vitro [128]. Furthermore, piezoelectric cardiac patches and hybrid electromechanical constructs, evaluated in porcine MI models, have been shown to preserve wall thickness and reduce infarct expansion, although clinical feasibility studies have not been performed [126].

Overall, the application of piezoelectric scaffolds offers several regenerative benefits, such as (a) promotion of cardiomyocyte growth, differentiation, and maturation; (b) enhancement of post‐MI tissue regeneration [127]; and (c) stimulation of angiogenesis, supporting vascularization of the damaged area, among others (Figure 6b, c) [127]. Thus, by delivering synchronized electrical and mechanical cues, piezoelectric scaffolds facilitate the formation of new, functional cardiac tissue, presenting a transformative approach for postinfarction cardiac repair.

Complementing these approaches, heart‐on‐a‐chip (HoC) platforms have emerged as essential tools for studying cardiac mechanobiology. These systems enable advanced modeling of cardiac function and provide robust platforms for investigating cardiovascular diseases, including cardiomyopathies, MI, ischemia/reperfusion injury, and fibrosis, as well as for drug screening and therapeutic development [129, 130].

HoC systems integrate both biochemical and mechanical cues, enabling the recreation of the dynamic 3D cardiac microenvironment. In particular, they replicate key physiological stimuli, including cyclic mechanical stretch, electrical pacing, and fluid‐induced shear stress, thereby closely mimicking native cardiac function. As HoC technology continues to advance, its physiological relevance and potential for translating mechanobiology‐driven regenerative strategies are increasingly strengthened.

However, despite these promising capabilities, several limitations remain. These include (a) challenges in reproducibility arising from variations in fabrication techniques and cardiac tissue culture methods; (b) the relative immaturity of human‐induced pluripotent stem cell–derived cardiomyocytes (hiPSC‐CMs), which currently do not fully recapitulate the functional complexity of adult cardiomyocytes; (c) limited accessibility due to the technical expertise required for device fabrication and operation; and (d) high costs, as the limited adoption of HoC systems restricts economies of scale, making them significantly more expensive than conventional 2D cultures and animal models [131]. Moreover, while in‐house fabrication is possible, it demands substantial investment in time, expertise, and resources [131].

### 5.4. Skin Regeneration and Topographically Guided Healing

**FIGURE 7:**
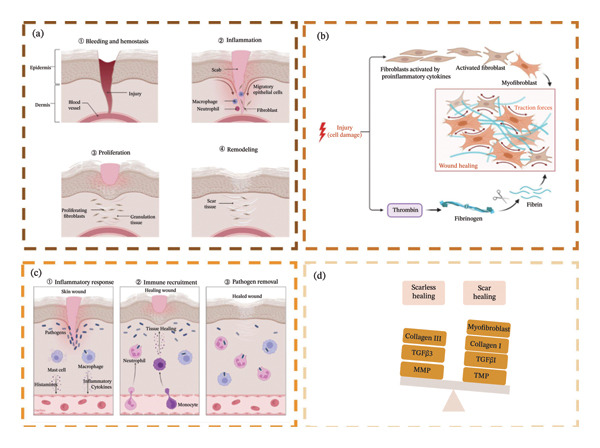
Sequential phases and cellular mechanisms of skin wound healing. (a) The four overlapping stages of wound healing—hemostasis, inflammation, proliferation, and remodeling—show the coordinated action of platelets, inflammatory cells, fibroblasts, and epithelial cells in restoring tissue integrity. (b) Role of fibrin deposition in activating fibroblasts and their differentiation into contractile myofibroblasts, generating traction forces essential for wound closure. (c) Immunological regulation of wound healing through inflammatory response, immune recruitment, and pathogen removal, highlighting the transition from inflammation to tissue repair. (d) Comparison between scar‐free and scar‐based healing outcomes. Scarless repair is characterized by higher expression of collagen III, TGF‐β3, and matrix metalloproteinases (MMPs), whereas fibrotic healing involves myofibroblast activation, collagen I deposition, and increased TGF‐β1 and TIMP expression. Adapted with permission [133].

The skin, the largest organ of the human body, serves as a critical barrier against environmental threats, regulates temperature, and minimizes water loss [132]. However, conventional wound healing often leads to scar formation, compromising both function and esthetics. Topographically guided healing offers a novel approach to improve skin regeneration while minimizing scarring. For instance, topographically patterned biomaterials (such as grooves, ridges, and micropatterned matrices) modulate fibroblast alignment, migration, and ECM deposition in in vitro wound healing platforms [93, 97]. The result is improved skin texture restoration and reduced scar visibility, providing outcomes closer to natural, uninjured skin. While this technique represents a significant advance in skin tissue engineering, enabling both functional and esthetic repair through biomechanical and biophysical modulation of the healing process (Figure 7), emerging skin‐on‐a‐chip (SoC) technologies offer an additional complementary approach for studying skin regeneration. These platforms are capable of recreating key features of the skin microenvironment, including its multilayered architecture and dynamic mechanical conditions, such as tensile stretching, compression, and fluid‐induced shear stress [134].

Consequently, SoC systems provide a promising framework for modeling wound healing processes across all stages (hemostasis, inflammation, proliferation, and remodeling), as well as for disease modeling and drug screening.

At the cellular level, skin is continuously subjected to mechanical forces, including compression, tension, and shear, arising from cell–cell interactions, ECM constraints, and cytoskeletal contractility, all of which play critical roles in regulating tissue function and repair [134].

In this context, SoC platforms enable controlled investigation of these mechanobiological cues under physiologically relevant conditions. More broadly, SoC technologies have rapidly advanced as biomimetic systems that address several limitations of conventional animal models by providing reproducible and human‐relevant platforms for ex vivo modeling of dermatological conditions [135].

Through precise control of the cellular microenvironment and the application of defined mechanical stimuli, these models have demonstrated improved barrier function, enhanced vascularization, and more physiologically relevant cellular differentiation compared to traditional in vitro systems [136].

**FIGURE 8:**
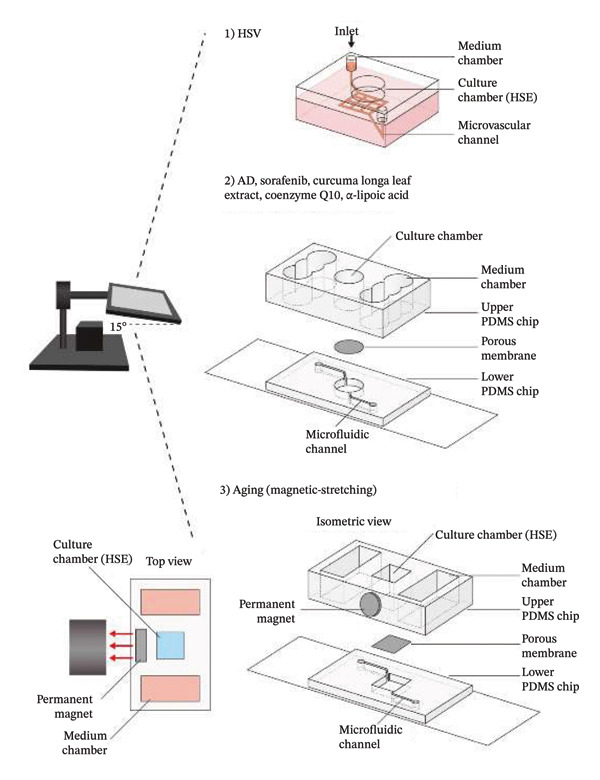
Schematic diagram of the skin‐on‐a‐chip and gravity flow system in action. A brief overview of different skin‐on‐a‐chip models to demonstrate various skin conditions and therapeutic agent effects. AD, atopic dermatitis; HSE, human skin equivalent; PDMS, polydimethylsiloxane. Adapted from [135] under CC BY 4.0 license.

Recent developments have expanded the application of SoC models across a wide range of areas, including infectious skin diseases (e.g., herpes simplex virus), inflammatory disorders (such as atopic dermatitis, psoriasis, and alopecia), and anti‐aging research (Figure 8) [135].

However, despite these advances, SoC technology is not yet a complete replacement for animal models. Key limitations include (a) the inability of a single platform to fully replicate all structural and functional components of skin, such as hair follicles, sweat glands, sensory receptors, and vascular networks, and (b) the challenge of capturing systemic, multi‐organ interactions that influence skin physiology and pathology [135].

As SoC systems primarily focus on localized skin environments, reproducing whole‐body biological complexity remains difficult; therefore, future mechanobiology‐mediated in situ tissue engineering strategies that incorporate SoC platforms should aim to address these limitations to enhance their clinical translatability and overall effectiveness.

### 5.5. Nerve Regeneration: Shape Memory Conduits and Electromechanical Stimulation

**FIGURE 9:**
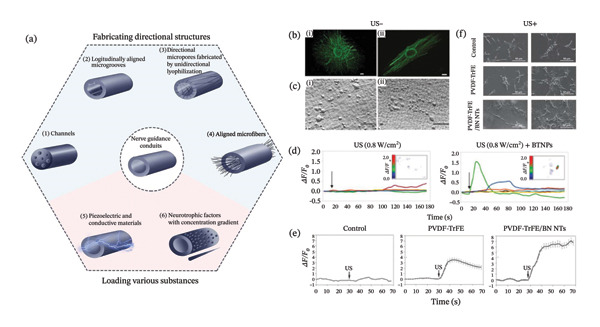
Strategies and electromechanical mechanisms for enhanced peripheral nerve regeneration. (a) Schematic illustration of principal design strategies for nerve guidance conduits (NGCs), including channelized or microgrooved architectures, unidirectional micropores, aligned microfibers, piezoelectric or conductive composites, and conduits loaded with neurotrophic factors or gradient cues to guide axonal regrowth. Adapted with permission from [137]. (b) Confocal fluorescence images of dorsal root ganglion (DRG) cells cultured on (i) random and (ii) aligned PVDF scaffolds showing neurite orientation along the fiber axis (phalloidin staining, scale bar = 300 μm). (c) Morphology of Nb2a neuroblastoma cells on (i) poled and (ii) unpoled PVDF substrates, demonstrating enhanced directional growth under poled conditions (scale bar = 100 μm). (d–e) Representative Ca^2+^‐imaging traces of SH‐SY5Y neuroblastoma cells stimulated with 0.8 W cm^−2^ ultrasound (US) showing transient intracellular Ca^2+^ flux, with amplified response in PVDF‐TrFE/BT nanoparticle composites. Arrows indicate 5‐s US pulse initiation. (f) SEM images of SH‐SY5Y cells cultured on control Ibid, PVDF‐TrFE, and PVDF‐TrFE/BT nanoparticle films under US (−/+) stimulation, highlighting improved cellular extension and alignment with piezoelectric activation. Adapted with permission from [138].

Peripheral nerve injuries often result in incomplete regeneration due to the absence of directional cues, leading to impaired nerve function and long‐term disability [133]. To address this challenge, recent research has introduced shape memory nerve guidance conduits (NGCs), advanced biomaterial tubular structures designed to bridge nerve gaps and support axonal regeneration (Figure 9) [137]. NGCs utilize shape memory polymers and electrically conductive materials to replicate the biomechanical and bioelectrical environment of native nerve tissue. Shape memory polymers provide mechanical integrity and structural support, allowing the conduit to conform to nerve defects while guiding regenerating axons along the correct path. This directional guidance is critical for restoring functional connections and improving the speed and quality of nerve repair. For instance, piezoelectric and conductive NGCs have been shown to regulate Schwann cell alignment, neuroblastoma cell polarization, and calcium flux in in vitro mechanotransduction studies [137, 139].

In vivo animal studies have demonstrated that electrical stimulation accelerates nerve recovery and promotes axon elongation, contributing to improved functional outcomes. For instance, in rodent sciatic nerve‐gap model studies, conductive and piezoelectric conduits accelerate axonal regrowth, increase myelination, and improve functional recovery metrics, such as SFI scores and CMAP amplitudes [138–140]. Additionally, piezoelectric materials, such as PVDF, have been shown to convert mechanical deformation into electrical signals, creating localized electromechanical stimulation that further enhances nerve regeneration. PVDF‐based conduits have successfully promoted peripheral nerve regeneration in mice, demonstrating their potential for clinical translation.

Recent advances in nerve‐on‐a‐chip (NoC) platforms have enabled the integration of controlled mechanical strain, electrical stimulation, and biochemical gradient cues to guide axonal growth and recapitulate patient‐specific pathophysiology. Using microfluidic technologies, these systems recreate the dynamic 3D neuronal microenvironment, offering physiologically relevant models for neural function and disease. For example, microfluidic NoC platforms have been applied in the development of diagnostic and therapeutic strategies for neurodegenerative diseases, including Alzheimer’s disease, Parkinson’s disease, amyotrophic lateral sclerosis, vascular dementia, and Huntington’s disease [141].

These platforms enable compartmentalization of neuronal cell bodies and axons, facilitating precise investigation of axon guidance and mechanotransduction pathways, such as calcium signaling and cytoskeletal remodeling.

Microfluidic organoid‐on‐chip systems have provided new opportunities to study brain development and neurophysiology by replicating the complex cellular interactions within the neurovascular unit. These systems offer fine control over biochemical and biophysical cues, continuous perfusion, and dynamic cell–cell interactions, thereby enhancing the physiological relevance of in vitro neural models [142].

Despite their considerable promise, several challenges remain. These include (a) difficulties in scaling up systems to accurately represent human tissue complexity, (b) the need to establish standardized protocols for quality control and functional validation, and (c) limitations in recreating highly integrated, multi‐organ interactions [142].

Furthermore, the generation of vascularized brain organoids remains resource‐intensive and less accessible compared to nonvascularized constructs, limiting widespread application. Advancing automation, scalability, and system integration will therefore be essential to fully realize the potential of these platforms in disease modeling and drug discovery [143].

Overall, these emerging NoC and organoid‐on‐chip technologies provide important tool kits to inform the design and optimization of NGCs and enhance mechanobiology‐driven in situ nerve regeneration strategies.

## 6. Conclusion and Future Outlooks

Mechanobiology‐mediated in situ tissue engineering promises to revolutionize the future of tissue engineering by recruiting the body’s own endogenous stem and progenitor cells to an injury site using biomaterials, 3D scaffolds, and bioactive molecules. Its potential (through preclinical and early clinical studies) across diverse tissue systems has been demonstrated. In orthopedics, for example, mechanically optimized 3D titanium‐mesh scaffolds have significantly enhanced bone regeneration and osseointegration, surpassing conventional grafts in load‐bearing defect models [140]. Similarly, in cardiovascular, neural, and dermal applications, smart hybrid scaffolds that integrate dynamic biomechanical cues have improved angiogenesis and functional integration [120]. Structurally biomimetic 3D‐printed scaffolds with hierarchical porosity and tuned mechanical performance are also achieving superior healing outcomes by promoting vascularization and mechanical stability [144]. These advances underscore the readiness of mechanobiology‐driven strategies for translation into clinical practice.

However, despite these advances, several key translational challenges remain. A major limitation involves the immune compatibility and safety of the biomaterial scaffolds designed to sense, transmit, or adapt to mechanical forces (mechanically responsive) within the physiological environment. Although such scaffolds can enhance tissue regeneration by replicating native biomechanical cues, their dynamic behavior and complex material composition may trigger inflammatory responses or unpredictable degradation in vivo. For instance, while preclinical successes have been demonstrated in controlled models, their long‐term safety and performance in diverse patient populations remain uncertain. Mechanical mismatch between scaffolds and native tissues can lead to poor load distribution, fibrosis, or implant failure. Additionally, the issue of incomplete vascularization and innervation of engineered constructs limits nutrient delivery and integration (particularly in large or complex tissue defects). Also, regulatory pathways for biomaterial 3D scaffolds and mechanically responsive implants are still evolving. Most tissue engineering scaffolds are recognized as a combination of two or more components (drug‐device, device‐biologic, drug‐biologic, or drug‐device‐biologic system), lacking proper standardization and creating uncertainty for clinical approval and commercialization. Finally, it is important from the ethical, regulatory, and economic standpoints that advances in mechanobiology‐mediated in situ tissue engineering, not only be standardized to receive early regulatory approval, but also (a) be compatible with sterilization platforms (gamma irradiation, ethylene oxide, steam, etc.), (b) affordable and cost‐effective, and (c) equitably accessed for quick clinical adoption.

Looking forward, in situ tissue engineering will benefit from next‐generation adaptive biomaterials, such as 4D scaffolds, that dynamically change shape or stiffness in response to physiological stimuli [144]. Integration of organoid‐on‐chip and microfluidic technologies, with implantable sensors and mechanostimulation devices, will enable closed‐loop systems, where mechanical cues and scaffold responses co‐evolve with patient healing. Additionally, advances in AI‐driven modeling, advanced imaging, and patient‐specific biomechanical profiling could arguably accelerate the development of mechanobiology‐mediated next‐generation in situ engineered personalized regenerative therapies [120, 145].

Ultimately, future research should focus on developing complex, multifunctional scaffolds that replicate the structural and dynamic properties of the native ECM. These scaffolds must not only direct cell behavior and growth factor release but also provide real‐time biological feedback, enabling precise and adaptive control of the healing process. By addressing current limitations, mechanobiology‐driven in situ tissue engineering has the potential to revolutionize regenerative medicine, bridging the gap between experimental success in research laboratories and clinically viable, next‐generation therapies.

## Funding

This work was supported by the Cambridge‐Africa Alborada Research Fund (grant numbers G1322276‐NMZG333).

## Conflicts of Interest

The authors declare no conflicts of interest.

## Data Availability

Data sharing is not applicable to this article as no datasets were generated or analyzed during the current study.
